# Qualitative thematic analysis based on descriptive phenomenology

**DOI:** 10.1002/nop2.275

**Published:** 2019-04-07

**Authors:** Annelie J. Sundler, Elisabeth Lindberg, Christina Nilsson, Lina Palmér

**Affiliations:** ^1^ Faculty of Caring Science, Work Life and Social Welfare University of Borås Borås Sweden

**Keywords:** healthcare research, lifeworld, lived experiences, meanings, midwifery, nursing, phenomenology, qualitative, thematic analysis

## Abstract

**Aim:**

The aim of this paper was to discuss how to understand and undertake thematic analysis based on descriptive phenomenology. Methodological principles to guide the process of analysis are offered grounded on phenomenological philosophy. This is further discussed in relation to how scientific rigour and validity can be achieved.

**Design:**

This is a discursive article on thematic analysis based on descriptive phenomenology.

**Results:**

This paper takes thematic analysis based on a descriptive phenomenological tradition forward and provides a useful description on how to undertake the analysis. Ontological and epistemological foundations of descriptive phenomenology are outlined. Methodological principles are explained to guide the process of analysis, as well as help to understand validity and rigour. Researchers and students in nursing and midwifery conducting qualitative research need comprehensible and valid methods to analyse the meaning of lived experiences and organize data in meaningful ways.

## 1 INTRODUCTION

Qualitative research in health care is an increasingly complex research field, particularly when doing phenomenology. In nursing and midwifery, qualitative approaches dealing with the lived experiences of patients, families and professionals are necessary. Today, there are number of diverse research approaches. Still, the clarity regarding approaches for thematic analysis is not yet fully described in the literature and only a few papers describe thematic analysis (Ho, Chiang, & Leung, 2017; Vaismoradi, Turunen, & Bondas, 2013). It may be difficult to find a single paper that can guide researchers and students in doing thematic analysis in phenomenology.

From our research experiences, it may be complex to read and understand phenomenological approaches. Similarly, the process of analysis can be challenging to comprehend. This makes methodological issues related to the clarity of ontological and epistemological underpinnings and discussions of validity and rigour complex. Norlyk and Harder (2010) points to difficulties finding a guide for phenomenological research. There is a need for understandable guidelines to take thematic analysis forward. Useful approaches are required to provide researchers and students guidance in the process of thematic analysis. With this paper, we hope to clarify some important methodological stances related to the thematic analysis of meaning from lived experiences that are grounded in descriptive phenomenology and useful to teachers and researchers in nursing and midwifery.

### 1.1 Background

Phenomenology has been widely used to understand human phenomena in nursing and midwifery practices (Matua, 2015). Today, there are several phenomenological approaches available. When using phenomenology, the researcher needs an awareness of basic assumptions to make important methodological decisions. Thus, it is important to understand the underpinnings of the approach used (Dowling & Cooney, 2012). Phenomenological underpinnings may, however, be difficult to understand and apply in the research process.

Thematizing meaning has been emphasized as one of a few shared aspects across different qualitative approaches (Holloway & Todres, 2003), suggesting that some qualitative research strategies are more generic than others. Although different approaches sometimes overlap, they have different ontological and epistemological foundations. A range of approaches are used to thematize meaning, but some of them would benefit from clarifying ontological and epistemological assumptions. In hermeneutic phenomenological traditions, thematizing meaning can be understood as related to the interpretation of data, illuminating the underlying or unspoken meanings embodied or hidden in lived experiences (Ho et al., 2017; van Manen, 2016). Another commonly used approach to thematic analysis is the method presented in the psychology literature by Braun and Clarke (2006). The method is frequently used to find repeated patterns of meaning in the data. However, there is a lack of thematic analysis approaches based on the traditions of descriptive phenomenology.

Researchers must make methodological considerations. In phenomenology, an awareness of the philosophical underpinning of the approach is needed when it is used in depth (Dowling & Cooney, 2012; Holloway & Todres, 2003). This places demands on methods to be comprehensible and flexible yet consistent and coherent. Questions remain regarding how thematic analysis can be further clarified and used based on descriptive phenomenology.

In this discursive paper, we provide guidance for thematic analysis based on descriptive phenomenology, which, to our knowledge, has not been made explicit in this way previously. This can be used as a guiding framework to analyse lived experiences in nursing and midwifery research. The aim of this paper was to discuss how to understand and undertake thematic analysis based on descriptive phenomenology. Methodological principles to guide the process of analysis are offered grounded on phenomenological philosophy. This is further discussed in relation to how scientific rigour and validity can be achieved.

## 2 ONTOLOGICAL AND EPISTEMOLOGICAL FOUNDATIONS OF DESCRIPTIVE PHENOMENOLOGY

Phenomenology consists of a complex philosophical tradition in human science, containing different concepts interpreted in various ways. One main theme among phenomenological methods is the diversity between descriptive versus interpretive phenomenology (Norlyk & Harder, 2010). Both traditions are commonly used in nursing and midwifery research. Several phenomenological methods have been recognized in the descriptive or interpretative approaches (Dowling, 2007; Dowling & Cooney, 2012; Norlyk & Harder, 2010). The descriptive tradition of phenomenology originated from the writings of Husserl was further developed by Merleau‐Ponty, while the interpretive approach was developed mainly from Heidegger and Gadamer.

The thematic analysis in this paper uses a descriptive approach with focus on lived experience, which refers to our experiences of the world. The philosophy of phenomenology is the study of a phenomenon, for example something as it is experienced (or lived) by a human being that means how things appear in our experiences. Consequently, there is a strong emphasis on lived experiences in phenomenological research (Dowling & Cooney, 2012; Norlyk & Harder, 2010). In this paper, lived experience is understood from a lifeworld approach originating from the writing of Husserl (Dahlberg, Dahlberg, & Nyström, 2008). The lifeworld is crucial and becomes the starting point for understanding lived experiences. Hence, the lifeworld forms the ontological and epistemological foundation for our understanding of lived experiences. In the lifeworld, our experiences must be regarded in the light of the body and the lifeworld of a person (i.e., our subjectivity). Consequently, humans cannot be reduced to a biological or psychological being (Merleau‐Ponty, 2002/1945). When understanding the meaning of lived experiences, we need to be aware of the lifeworld, our bodily being in the world and how we interact with others.

The understanding of lived experiences is closely linked to the idea of the intentionality of consciousness, or how meaning is experienced. Intentionality encompasses the idea that our consciousness is always directed towards something, which means that when we experience something, the “thing” is experienced as “something” that has meaning for us. For example, a birthing woman's experience of pain or caregiving as it is experienced by a nurse. In a descriptive phenomenological approach, based on the writing of Husserl (Dahlberg et al., 2008) such meanings can be described. From this point of view, there are no needs for interpretations of these meanings, although this may be argued differently in interpretive phenomenology. Intentionality is also linked to our natural attitude. In our ordinary life, we take ourselves and our life for granted, which is our natural attitude and how we approach our experiences. We usually take for granted that the world around us is as we perceive it and that others perceive it as we do. We also take for granted that the world exists independently of us. Within our natural attitude, we normally do not constantly analyse our experiences. In phenomenology, an awareness of the natural attitude is important.

## 3 METHODOLOGICAL PRINCIPLES

In the ontological and epistemological foundations of descriptive phenomenology, some methodological principles can be recognized and how these are managed throughout the research process. Phenomenological studies have been criticized for lacking in clarity on philosophical underpinnings (Dowling & Cooney, 2012; Norlyk & Harder, 2010). Thus, philosophical stances must be understood and clarified for the reader of a study. Our suggestion is to let the entire research process, from data gathering to data analysis and reporting the findings, be guided by the methodological principles of *emphasizing openness*, *questioning pre‐understanding* and adopting a *reflective attitude*. We will acknowledge that the principles presented here may not be totally distinct from, or do follow, a particular phenomenological research approach. However, the outlined approach has some commonalities with the approaches of, for example, Dahlberg et al. (2008) and van Manen (2016).

When researching lived experiences, *openness* to the lifeworld and the phenomenon focused on must be emphasized (i.e., having curiosity and maintaining an open mind when searching for meaning). The researcher must adopt an open stance with sensitivity to the meaning of the lived experiences currently in focus. Openness involves being observant, attentive and sensitive to the expression of experiences (Dahlberg et al., 2008). It also includes questioning the understanding of data (Dahlberg & Dahlberg, 2003). Thus, researchers must strive to maintain an attitude that includes the assumption that hitherto the researcher does not know the participants experience and the researcher wants to understand the studied phenomenon in a new light to make invisible aspects of the experience become visible.

When striving for openness, researchers need to *question their pre‐understanding*, which means identifying and becoming aware of preconceptions that might influence the analysis. Throughout the research process and particularly the analysis, researchers must deal with the natural attitude and previous assumptions, when analysing and understanding the data. Questioning involves attempting to set aside one's experiences and assumptions as much as possible and means maintaining a critical stance and reflecting on the understanding of data and the phenomenon. This is similar to bracketing, a commonly used term in descriptive phenomenology based on Husserl, but it has been criticized (Dowling & Cooney, 2012). Some would argue that bracketing means to put aside such assumptions, which may not be possible. Instead, Gadamer (2004) deals with this in a different way, arguing that such assumptions are part of our understanding. Instead of using bracketing, our intention is to build on questioning as a representative way to describe what something means. Accordingly, researchers need to recognize personal beliefs, theories or other assumptions that can restrict the researcher's openness. Otherwise, the researcher risks describing his or her own pre‐understanding instead of the participants' experiences. Our pre‐understanding, described as “prejudice” in interpretive phenomenology by Gadamer (2004), is what we already know or think we know about a phenomena. As humans, we always have such a pre‐understanding or prejudice and Gadamer (2004) posits this is the tradition of our lived context and emphasizes that our tradition has a powerful influence on us. This means that it might be more difficult to see something new in the data than describe something already known by the researcher. Therefore, an open and sensitive stance is needed towards oneself, one's pre‐understanding and the understanding of data. However, one must be reflective and critical towards the data, as well as how to understand meanings from the data. Questioning can help researchers become aware of their pre‐understanding and set aside previous assumptions about the phenomenon (Dahlberg et al., 2008).

Questioning one's pre‐understanding is closely linked to having a *reflective attitude*. With a reflective attitude, the researcher needs to shift from the ordinary natural understanding of everyday life to a more self‐reflective and open stance towards the data (Dahlberg et al., 2008). An inquiring approach throughout the research process helps researchers become more aware of one's assumptions and reflect regarding the context of the actual research. For instance, researchers may need to reflect on why some meanings occur, how meanings are described and if meanings are grounded in the data. In striving for an awareness of the natural attitude, a reflective attitude becomes imperative. By having such an awareness, some of the pitfalls related to our natural attitude can be handled in favour of an open and reflective mind.

To summarize, methodological principles have been described in terms of emphasizing openness, questioning pre‐understanding and adopting a reflective attitude, which are three related concepts. To emphasis openness, one needs to reflect on preconceptions and judgements concerning the world and our experiences with a reflective approach to become aware of the natural attitude and process of understanding. Engaging in critical reflection throughout the research process may facilitate an awareness of how the researcher influences the research process. These methodological principles, related to ontological and epistemological foundations of phenomenology, are suggested to guide the research process, particularly the analysis.

## 4 THEMATIC ANALYSIS OF LIVED EXPERIENCES

The thematic analysis approach described in this paper is inductive. A prerequisite for the analysis is that it includes data on lived experiences, such as interviews or narratives. Themes derived from the analysis are data driven (i.e., grounded in data and the experience of the participants). The analysis begins with a search for meaning and goes on with different meanings being identified and related to each other. The analysis is aimed to try to understand the complexity of meanings in the data rather than measure their frequency. It involves researcher engaging in the data and the analysis. The analysis contains a search for patterns of meanings being further explored and determining how such patterns can be organized into themes. Moreover, the analysis must be guided by openness. Thus, the analysis involves a reflective process designed to illuminate meaning. Although the process of analysis is similar to descriptive phenomenological approaches focusing on the understanding and description of meaning‐oriented themes (Dahlberg et al., 2008; van Manen, 2016), there are important differences. While the thematic analysis in this paper focuses on how to organize patterns of meaning into themes, some would argue that an essential, general structure of meaning, rather than fragmented themes, is preferred (van Wijngaarden, Meide, & Dahlberg, 2017) and that such an essential meaning structure is a strength. We argue that meaning‐oriented themes can contribute to robust qualitative research findings. Still, it is important that the findings move between concrete expressions and descriptive text on meanings of lived experiences.

### 4.1 The process of analysis

**Figure 1:**
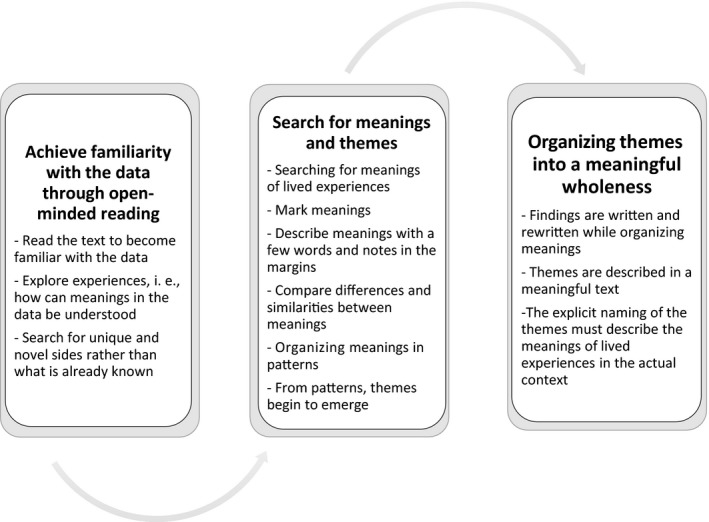
Summary of thematic analysis

The goal of the thematic analysis is to achieve an understanding of patterns of meanings from data on lived experiences (i.e., informants' descriptions of experiences related to the research question in, e.g., interviews or narratives). The analysis begins with data that needs to be textual and aims to organize meanings found in the data into patterns and, finally, themes. While conducting the analysis, the researcher strives to understand meanings embedded in experiences and describe these meanings textually. Through the analysis, details and aspects of meaning are explored, requiring reading and a reflective writing. Parts of the text need to be understood in terms of the whole and the whole in terms of its parts. However, the researcher also needs to move between being close to and distant from the data. Overall, the process of analysis can be complex and the researcher needs to be flexible. This process is summarized in Figure 1 and detailed in the description below.

To begin the analysis, the researcher needs to achieve familiarity with the data through open‐minded reading. The text must be read several times in its entirety. This is an open‐ended reading that puts the principle of openness into practice with the intention of opening one's mind to the text and its meanings. When reading, the researcher starts to explore experiences expressed in the data, such as determining how these are narrated and how meanings can be understood. The goal is to illuminate novel information rather than confirm what is already known while keeping the study aim in mind.

Thereafter, the parts of the data are further illuminated and the search for meanings and themes deepens. By moving back and forth between the whole and its parts, a sensitive dialogue with the text may be facilitated. While reading, meanings corresponding to the study's aim are marked. Notes and short descriptive words can be used to give meanings a preliminary name. As the analysis progresses, meanings related to each other are compared to identify differences and similarities. Meanings need to be related to each other to get a sense of patterns. Patterns of meanings are further examined. It is important to not make meanings definite too rapidly, slow down the understanding of data and its meanings. This demands the researcher's openness to let meanings emerge.

Lastly, the researcher needs to organize themes into a meaningful wholeness. Methodological principles must remind the researcher to maintain a reflective mind, while meanings are further developed into themes. Meanings are organized into patterns and, finally, themes. While deriving meaning from text, it is helpful to compare meanings and themes derived from the original data. Nothing is taken for granted, and the researcher must be careful and thoughtful during this part of the process. It can be valuable to discuss and reflect on tentative themes emerging from the data. Findings need to be meaningful, and the naming and wording of themes becomes important. The writing up of the themes is aimed to outline meanings inherent in the described experiences. At this point, findings are written and rewritten. Faithful descriptions of meanings usually need more than a single word, and the writing is important.

To conclude, the process of thematic analysis, based in a descriptive phenomenological approach, goes from the original data to the identification of meanings, organizing these into patterns and writing the results of themes related to the study aim and the actual context. When the findings are reported, these are described conversely (i.e., starting with the themes and the descriptive text, illustrated with quotes). Thus, meanings found from participants experiences are described in a meaningful text organized in themes.

### 4.2 Validity and Rigour

Hereby follows our discussion on scientific quality in terms of validity and rigour in the thematic analysis process. There is no consensus on which concepts should be used regarding validity in qualitative and phenomenological research. The term validity is typically used in relation to quantitative methods; however, qualitative researchers claim that the term is suitable in all paradigms as a generic term implying whether the research conclusions are sound, just and well‐founded (Morse, 2015; Whittemore, Chase, & Mandle, 2001). Rolfe (2006) states that scientific rigour can be judged based on how the research is presented for the reader and appraising research lies with both the reader and the writer of the research. Thus, clarity regarding methodological principles used becomes necessary. Porter (2007) argues that a more realistic approach is needed and that scientific rigour needs to be taken seriously in qualitative research (Porter, 2007). It has been stressed that strategies are needed to ensure rigour and validity; such strategies must be built into the research process and not solely evaluated afterwards (Cypress, 2017). Therefore, we further discuss scientific rigour and phenomenological validity in relation to *reflexivity*, *credibility* and *transferability*.

*Reflexivity* is strictly connected to previously described methodological principles of a reflective attitude and questioning one's pre‐understanding. Reflexivity must be maintained during the entire process, and the researcher needs to sustain a reflective attitude. Particularly, reflexivity must involve questioning the understanding of data and themes derived. Qualitative researchers are closely engaged in this process and must reflect on what the data actually state that may be different from the researcher's understanding. This means the researcher should question the findings instead of taking them for granted. Malterud (2001) claims that multiple researchers might strengthen the study since they can give supplementary views and question each other's statements, while an independent researcher must find other strategies. Another way to maintain reflexivity is comparing the original data with the descriptive text of themes derived. Moreover, findings need to be illustrated with original data to demonstrate how the derived descriptions are grounded in the data rather than in the researcher's understanding. Furthermore, information is needed on the setting so the reader can understand the context of the findings.

*Credibility* refers to the meaningfulness of the findings and whether these are well presented (Kitto, Chesters, & Grbich, 2008). Credibility and reflexivity are not totally distinct but are correlated with each other. Credibility stresses that nothing can be taken for granted and is associated with the methodological principles described above. The researcher needs to emphasize how the analysis and findings are presented for the reader. The analysis needs to be transparent, which means that the researcher should present it as thoroughly as possible to strive for credibility. The reader needs information concerning the methodology used and methodological decisions and considerations made. This includes, for instance, how the thematic analysis was performed, descriptions of how meanings were derived from the data and how themes were identified. Descriptions need to be clear and consistent. However, it must be possible to agree with and understand the logic of the findings and themes. Credibility lies in both the methodology and in the presentation of findings. Thus, in striving for credibility, the procedures and methods need to be presented as thoroughly and transparently as possible. Themes described must be illustrated with quotes to ensure the content and described meanings are consistent.

*Transferability* refers to the usefulness and relevance of the findings. However, the method used does not guarantee transferability in itself. Transferability is not explicitly related to any of the methodological principles, but it may be a result of them. Transferability is a measure of whether the findings are sound and if the study adds new knowledge to what is already known. The clarity of findings is also important. Thus, findings must be understandable and transferable to other research (i.e., findings need to be recognizable and relevant to a specific or broader context other than the original study). Specifically, the relevance, usefulness and meaningfulness of research findings to other contexts are important components of the study's transferability.

**Figure 2:**
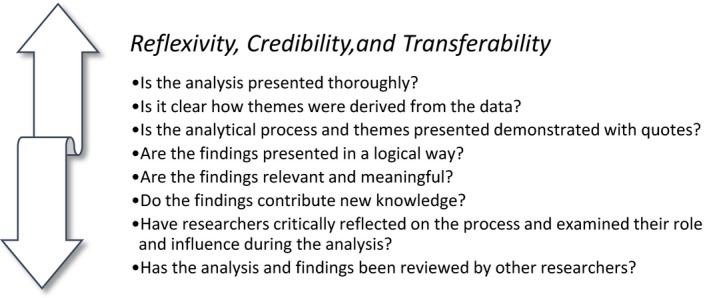
Overview of questions useful to the uphold reflexivity, credibility and transferability of the research process in the thematic analysis of meanings

To conclude, reflexivity, credibility and transferability are concepts important to acknowledge and consider throughout the research process to engender validity and rigour. We maintain that meaning‐oriented themes can contribute to robust findings, if reported in a text describing patterns of meanings illustrated with examples of expressions from lived experiences. Questions researchers need to ask themselves in relation to validity when conducting a thematic analysis are presented in Figure 2. Since the method in itself is no guarantee of validity and rigour, discussions related to these areas are needed.

## 5 IMPLICATIONS FOR NURSING AND MIDWIFERY

In this paper, a method for thematic analysis based on phenomenology has been outlined. Doing phenomenological research is challenging. Therefore, we hope this paper contributes to the understanding of phenomenological underpinnings and methodological principles of thematic analysis based on descriptive phenomenology. This approach can be useful for teachers and researchers in nursing and midwifery. The thematic analysis presented can offer guidance on how to understand meaning and analyse lived experiences. Methodological stances of descriptive phenomenology are clarified, linking the process of analysis with theoretical underpinnings. Methodological principles are explained to give guidance to the analysis and help understand validity and rigour. Thus, this paper has the potential to provide researchers and students who have an interest in research on lived experiences with a comprehensive and useful method to thematic analysis in phenomenology. Nurses and midwives conducting qualitative research on lived experiences need robust methods to ensure high quality in health care to benefit patients, childbearing women and their families.

## 6 CONCLUSION

We provide researchers in nursing and midwifery with some clarity regarding thematic analysis grounded in the tradition of descriptive phenomenology. We argue that researchers need to comprehend phenomenological underpinnings and be guided by these in the research process. In thematic analysis, descriptive phenomenology is a useful framework when analysing lived experiences with clarified applicable ontological and epistemological underpinnings. Emphasizing openness, questioning pre‐understanding and adopting a reflective attitude were identified as important methodological principles that can guide researchers throughout the analysis and help uphold scientific rigour and validity. For novice researchers, the present paper may serve as an introduction to phenomenological approaches.

## CONFLICT OF INTEREST

No conflict of interest has been declared by the authors.

## AUTHOR CONTRIBUTIONS

AS, EL, CN, LP: Made substantial contributions to conception and design, or acquisition of data, or analysis and interpretation of data; involved in drafting the manuscript or revising it critically for important intellectual content; given final approval of the version to be published and each author should have participated sufficiently in the work to take public responsibility for appropriate portions of the content; and agreed to be accountable for all aspects of the work in ensuring that questions related to the accuracy or integrity of any part of the work are appropriately investigated and resolved.
